# Polymorphisms of *T- cell leukemia 1A* gene loci are not related to the development of adjuvant letrozole-induced adverse events in breast cancer

**DOI:** 10.1371/journal.pone.0247989

**Published:** 2021-03-24

**Authors:** Gurusamy Umamaheswaran, Dharanipragada Kadambari, Suresh Kumar Muthuvel, Naveena A. N. Kumar, Biswajit Dubashi, Steven Aibor Dkhar, Chandrasekaran Adithan

**Affiliations:** 1 Department of Pharmacology, Centre for Advanced Research in Pharmacogenomics, Jawaharlal Institute of Postgraduate Medical Education and Research (JIPMER), Puducherry, India; 2 Departments of Surgery and Medical Education, Jawaharlal Institute of Postgraduate Medical Education and Research (JIPMER), Puducherry, India; 3 Center for Bioinformatics, School of Life Sciences, Pondicherry University, Puducherry, India; 4 Department of Medical Oncology, Regional Cancer Center, Jawaharlal Institute of Postgraduate Medical Education and Research (JIPMER), Puducherry, India; 5 Department of Clinical Pharmacology, Jawaharlal Institute of Postgraduate Medical Education and Research (JIPMER), Puducherry, India; King Saud University, SAUDI ARABIA

## Abstract

Letrozole, an aromatase inhibitor (AI), is the first-line adjuvant drug for treating hormone receptor-positive (HR+) breast cancer in postmenopausal women. However, harmful adverse events (AEs) and significant differences in drug response among individuals remain a significant problem in clinical application. Current evidence suggests that the observed individual variation in the treatment outcomes of AI is conferred by genetic variants. Hence, in this study, we examined the association of *TCL1A* gene polymorphisms with letrozole-induced AEs. The study subjects were postmenopausal HR+ breast cancer patients who were receiving adjuvant letrozole. Genomic DNA was isolated by a routine standard phenol-chloroform method. In total, 198 South Indian patients were genotyped for four single nucleotide polymorphisms (SNPs) in the *TCL1A* gene loci by the TaqMan allelic discrimination assay using the RT-PCR system. We used the odds ratio and 95% confidence interval to assess the genetic association. Musculoskeletal (MS) AEs and vasomotor symptoms (VMSs) are the most common side effects observed in the study cohort. Among 198 patients, 81 experienced musculoskeletal toxicity, reporting MS-AEs, 57 had VMSs, and 33 of them had both. The most frequently identified polymorphic variants in the patient series were *rs11849538 (G)*, with an allele frequency of about 27.3%, followed by *rs7158782-G* (27.3%), *rs7159713-G* (25.8%), and *rs2369049-G* (22.5%). The genetic association analysis indicated no significant difference in the proportion of *TCL1A* gene variants between patients with and without AEs on either MS-AEs or VMSs. Though we observed high LD in all patient groups, the inferred haplotypes displayed a non-significant association with letrozole-induced specific AEs. However, the SNP functionality analysis by RegulomeDB provided a 2b rank score for *rs7158782*, suggesting a potential biological function. Our findings suggest that *TCL1A* gene polymorphisms may not play any role in the prediction of letrozole-induced AEs in South Indian HR+ breast cancer patients.

## Introduction

Adverse drug reactions (ADRs) are harmful and undesirable effects on the human body resulting from pharmacological interventions [1]. ADRs cause serious health burdens and have become a significant contributor to the cost inflation in the healthcare system. These reactions can range from mild to potentially life-threatening adverse events (AEs). Susceptibility to ADRs can be due to many factors that include both genetic and non-genetic factors [2]. Despite significant advancements and breakthroughs in genetic determinants of ADRs, only a few isolated pharmacogenetic tests for ADR predisposition have been translated into clinical practice [3].

Letrozole, a non-steroidal third-generation aromatase inhibitor (AI), and a standard of care to postmenopausal women for the treatment of hormone receptor-positive (HR+) breast cancer [4]. This drug is proven to be effective over tamoxifen therapy in the context of disease-free survival and AEs. It has, therefore, been the most widely recommended first-line adjuvant drug worldwide [5,6]. Letrozole prevents the biosynthesis of estrogens, thereby lowering E1 and E2 levels and reducing the disease’s advancement and recurrence risk. Unfortunately, despite the advances as an anti-aromatase compound, high inter-individual variability in letrozole response and toxicity among patients remains a major problem. In clinical settings, women taking AIs have higher risks of musculoskeletal (MS) AEs, fractures, and enhanced bone loss. Indeed, 50% of the patients with AIs suffer from arthralgia resulting in reduced drug adherence and efficacy, necessitating early withdrawal of therapy [7]. Vasomotor symptoms (VMSs) are another well-known side effect of endocrine therapy administration that also occurs during menopause [8].

It has been hypothesized that the observed individual variation in treatment outcomes to this drug is conferred by genetic variants. A multitude of population-based molecular epidemiological studies has investigated the germline genetics of AIs [9–13]. These genetic variations, in particular, variants in the *CYP19A1* gene, have evolved as essential contributors to predict the inter-patient differences in drug effectiveness and toxicity after the administration of AIs; particularly, the non-steroidal agent letrozole. Accordingly, we have identified a significant association between *CYP19A1* variants and letrozole-induced AEs [14]. With different polymorphisms, treatment settings, endpoints, study design, and ethnic groups, these studies have yielded conflicting results. More recently, a genome-wide association study (GWAS) discovered genetic variants localized in the region of *TCL1A* gene loci appeared to influence the risk of MS-AEs in patients undergoing AIs treatment [15], suggesting that this gene plays a role for MS toxicity.

The gene *TCL1A (T Cell Leukemia/Lymphoma 1A)* encoding the 14 kDa TCL1A protein is located at chromosome 14q32.1. It is a member of the TCL1 family and expressed in a range of cells, in particular, activated T and B lymphocytes [16]. The knowledge about *TCL1A* gene properties was limited, as this gene had not been cloned. However, studies on the *TCL1A* gene implicated its possible involvement in various hematopoietic malignancies [17]. ADRs are among the common causes of morbidity and mortality in cancer patients and have therefore been the subject of intense research in cancer therapy. The effect of *TCL1A* polymorphisms in HR+ postmenopausal breast cancer patients treated with letrozole needs further research to confirm its effect on other independent populations. As evidenced by our earlier reports, the South Indians represent a unique set of ethnicity-specific genetic signatures within the pattern of genetic variants in genes that governs drug metabolism and transport as compared to other global races [18].

Till now, no research into *TCL1A* gene polymorphisms and AIs treatment outcomes has been described in Indians. Therefore, we indended to understand the clinical relevance of *TCL1A* genetic polymorphisms on the development of letrozole-induced ‘specific’ AEs in postmenopausal HR+ breast cancer patients of South Indian ancestry.

## Materials and methods

### Patient population

We identified a total of 198 breast cancer patients (median age 56 years with a range from 38 to 85 years) who were getting adjuvant treatment (Letrozole, 2.5mg OD) at Tumor Clinic, Outpatient Departments of General Surgery and Medical Oncology, Jawaharlal Institute of Postgraduate Medical Education and Research (JIPMER), Pondicherry from January 2009 through January 2014. The research protocol was approved by the Institutional Scientific Advisory and Research Ethics Committees of JIPMER, Pondicherry. Signed informed consent was obtained from each study subject. All of them were of South Indian origin, living in the southern states of Tamil Nadu, Puducherry, Karnataka, Kerala, Andhra Pradesh, and Telangana for more than three generations. The diagnosis of breast cancer was confirmed by histopathological examination. Details of this study design, selection criteria, toxicity assessment, questionnaire, and treatment have been described previously [14].

### DNA extraction and genotyping

We extracted the genomic DNA from stored peripheral blood mononucleated cells by the routine standard phenol-chloroform method and used as DNA templates for genotyping. Genotypes of rs7158782 (assay ID C_29078024_10), rs7159713 (assay ID C_1927662_10), rs2369049 (assay ID C_1927663_20), and rs11849538 (assay ID C_1927667_10), SNPs were determined with predesigned TaqMan SNP genotyping assays obtained from Life Technologies on ABI 7300 quantitative RT-PCR system based on allelic discrimination according to the manufacturer’s instructions. The details of the RT-PCR procedure have been described previously [19]. For quality control, replication was performed by other laboratory personnel in 30% of the randomly chosen samples to confirm the authenticity of the genotype results obtained earlier, and they were found to be in 100% concordance.

### Statistical analyses

Haplotype frequency and linkage disequilibrium (LD) patterns were analyzed using Haploview program version 4.2 (http://www.broad.tamit.edu/mpg/haploview). The allele and genotype frequencies were calculated by the direct gene count method. Hardy-Weinberg equilibrium (HWE) was achieved by the chi-square test to assess the bias in the genotype distribution. Patients were classified into five subgroups based on the presence or absence of ‘specific’ AEs: MS-AEs (patients with MS-AEs), VMSs (patients with VMSs), Both (patients with MS-AEs and VMSs), Any AEs (patients with either MS-AEs or VMSs), and No AEs (patients without any AEs). For the genetic association analysis, we applied logistic regression to estimate odds ratio (OR) and 95% confidential interval (CI) for each group vs. patients without any AEs using six different genetic models (allele, co-dominant, dominant, recessive, over-dominant, and additive). The covariates adjusted were age, type/regime of chemotherapy, reasons for menopause, and time since the menopause (LMP). The above statistical tests were carried out using SPSS version 19.0 (SPSS Inc., Chicago, IL, USA). All the p values were two-sided, and the statistical significance threshold was set at P< 0.05. To predict the functional significance of each SNP, we employed RegulomeDB (http://www.regulomedb.org) online database.

We calculated the sample size to be 198 using PS Power & Sample Size Calculations software version 3.1.2 and the QUANTO program. The power was set at 0.80; α = 0.05 based on the least variant allele frequency of *TCL1A* gene polymorphism (rs2369049-G allele, 21.2%) in healthy South Indians [19] to demonstrate an association between the variant genotypes and letrozole-related ‘specific’ adverse events. Further, genetic heterogeneity could lead to either reduced power or spurious associations in pharmacogenetic epidemiological studies. Hence, we carried out a population-specific investigation restricting to South Indian subjects to avoid the ethnicity variations leading to a pharmacogenetic anomaly.

## Results

### Baseline characteristic features of the study cohort

The baseline histopathological and clinical characteristics of all the study subjects are summarized and presented in Table 1. The median age of the series was 56 years, with a range from 38 to 85 years; the median BMI was 25.8 kg/m2 (range 14.8–45.2). As shown, most of the cases were grade I-II (83.8%), and most of them had a T2 tumor (38.9%). All the tumors expressed hormone receptor positivity, either ER +ve (89.9%) or PR +ve (67.2%) or both (55.5%), and 70 patients were positive for Her2. Among the diagnosed patients, chemotherapy was given to a total of 163 patients. Additionally, 80.8% of patients (160/198) had been treated with radiotherapy, and 22.2% of patients (44/198) completed prior tamoxifen therapy. Twenty-seven patients (13.6%) underwent BCS, and the remaining 171 cases had undergone a radical mastectomy. We have distinguished patients’ education status into two groups: those with a high school or lower education (43.9%) and those with college or higher studies (9.6%). Regarding menopause, 141 patients attained it naturally, and the rest 57 cases were induced either by surgery or chemotherapy. We previously reported the clinical and pathological characteristics of patients stratified by ‘specific’ adverse events for this patient cohort [14].

**Table 1:** Baseline characteristic features of patients in the study population.

Parameter	N (%)	Parameter	N (%)
Median age, years (range)	56 (38–85)	Chemotherapy	163 (82.3)
Median BMI, kg/m2 (range)	25.8 (14.8–45.2)	Prior tamoxifen	44 (22.2)
Median BSA, m2 (range)	1.54 (1.21–1.93)	Surgery	
Age at diagnosis	54 (31–81)	Mastectomy	171 (86.4)
Age at menarche	14 (11–19)	BCS	27 (13.6)
Age at menopause	45 (32–58)	Educational status	
Years of menstruation	31 (19–46)	No schooling	92 (46.5)
Family history of Ca breast	28 (14.2)	High school or lower	87 (43.9)
Histological grade		College or higher	19 (9.6)
I	70 (35.3)	Reason for menopause	
II	96 (48.5)	Natural	141 (71.2)
III	32 (16.2)	Induced	57 (28.8)
Tumor size		Year since last menopause	
T1	21 (10.6)	<5	50 (25.2)
T2	77 (38.9)	5 to 10	67 (33.8)
T3	52 (26.3)	>10	81 (41.0)
T4	48 (24.2)	Year since start of letrozole	
Lymph node involvement		<1	25 (12.6)
N0	74 (37.4)	1 to 3	103 (52.0)
N1	100 (50.5)	>3	70 (35.4)
N2	18 (9.1)	Other Illness	
N3	6 (3.0)	Diabetes	54 (27.3)
Hormone receptor status		Hypertension	59 (29.8)
ER positive	178 (89.9)	Asthma	9 (4.5)
PR positive	133 (67.2)	Concomitant Medication	
Her2/neu positive	70 (35.4)	Metformin	40 (20.2)
Tumour site		Amlodipine	30 (15.1)
Left breast	101 (51.0)	Glibenclamide	19 (9.6)
Right breast	94 (47.5)	Enalapril	18 (9.0)
Both	3 (1.5)	Atenolol	7 (3.5)
Radiotherapy	160 (80.8)	Salbutamol	4 (2.0)

N, number of subjects; BMI, body mass index; BSA, body surface area; BCS, breast conservation surgery; ER, Estrogen receptor; PR, progesterone receptor; Ca, cancer.

### Frequency distribution and functional annotation of *TCL1A* genetic polymorphisms

Table 2 describes the genotype and allele frequency distributions of *TCL1A* genetic variants in the study population. According to HWE, the observed genotype frequencies of all the tested genetic variants were in agreement with the expected frequencies for both patients with and without ‘specific’ AEs. The most frequently identified polymorphic variant in the patient series was rs11849538 (G), with an allele frequency of about 27.3%. The observed frequency of cases with other minor alleles were rs7158782-G (27.3%), rs7159713-G (25.8%), and rs2369049-G (22.5%). All the polymorphisms described in this study were evaluated with Regulome RD online portal for their in silico functional prediction. Of them, the SNP rs7158782 has a less score of 2b, indicates its potential functional significance.

**Table 2:** Observed genotype and allele frequency distributions in patients with and without adverse events.

SNP	RegulomeDB	Genotype & Allele	Patients (N = 198)	MS-AEs	VMSs	Both	Any AEs	No AEs
				(N = 81)	(N = 57)	(N = 33)	(N = 105)	(N = 93)
*rs7158782*	*2b*	*AA*	110 (55.6)	46 (56.8)	32(56.2)	19(57.6)	59(56.2)	51(54.8)
		*AG*	74 (37.4)	29 (35.8)	24(42.1)	13(39.4)	40(38.1)	34(36.6)
		*GG*	14 (7.0)	6 (7.4)	1(1.7)	1 (3.0)	6(5.7)	8(8.6)
		*A*	294 (74.2)	121 (74.7)	88(77.2)	51(77.3)	158(75.2)	136(73.1)
		*G*	102 (25.8)	41 (25.3)	26(22.8)	15(22.7)	52(24.8)	50(26.9)
*rs7159713*	*7*	*AA*	114 (57.6)	47 (58.0)	34(59.7)	19(57.6)	62(59.1)	52(55.9)
		*AG*	70 (35.4)	28 (34.6)	22(38.6)	13(39.4)	37(35.2)	33(35.5)
		*GG*	14 (7.0)	6 (7.4)	1(1.7)	1 (3.0)	6(5.7)	8(8.6)
		*A*	298 (75.3)	122 (75.3)	90(79.0)	51(77.3)	161(76.7)	137(73.7)
		*G*	98 (24.7)	40 (24.7)	24(21.0)	15(22.7)	49(23.3)	49(26.3)
*rs2369049*	*7*	*AA*	121 (61.1)	50 (61.7)	36(63.2)	21(63.6)	65(61.9)	56(60.2)
		*AG*	65 (32.9)	25 (30.9)	20(35.1)	11(33.4)	34(32.4)	31(33.3)
		*GG*	12 (6.0)	6 (7.4)	1(1.7)	1 (3.0)	6(5.7)	6(6.5)
		*A*	307 (77.5)	125 (77.2)	92(80.7)	53(80.3)	164(78.1)	143(76.9)
		*G*	89 (22.5)	37 (22.8)	22(19.3)	13(19.7)	46(21.9)	43(23.1)
*rs11849538*	*5*	*CC*	105 (53.0)	45 (55.6)	30(52.6)	19(57.6)	56(53.3)	49(52.7)
		*CG*	78 (39.4)	29 (35.8)	26 (45.6)	13(39.4)	42(40.0)	36(38.7)
		*GG*	15 (7.6)	7 (8.6)	1(1.7)	1 (3.0)	7(6.7)	8(8.6)
		*C*	288 (72.7)	119 (73.4)	86(75.4)	51(77.3)	154(73.3)	134(72.0)
		*G*	108 (27.3)	43 (26.6)	28(24.6)	15(22.7)	56(26.7)	52(28.0)

SNP, single nucleotide polymorphism; MS-AEs, musculoskeletal adverse events; VMSs, vasomotor symptoms; N, number of subjects; Both, patients with MS-AEs and VMSs, Any AEs, patients with either MS-AEs or VMSs; No AEs, patients without any AEs; Values in parentheses indicate percentage; 2b, TF binding + any motif + DNase Footprint + DNase peak; 5, TF binding or DNase peak; 7, other.

### Association between letrozole-induced ‘specific’ AEs and *TCL1A* genetic variants

To evaluate the genetic effects of *TCL1A* genotypes on MS toxicity risk, the distribution of the dataset for each SNP was analyzed for co-dominant, dominant, recessive, over-dominant, and additive genetic models (Table 3). None of the individual *TCL1A* genetic polymorphisms were found to be significantly associated with MS-AEs risk. As evident from Table 3, the association analyses of *TCL1A* genetic polymorphisms with VMSs also revealed statistically no significant difference (P>0.05) in the proportion of the genotype frequency among patients with VMSs and without any AEs. The different genetic model predictions also displayed a non-significant association with VMSs risk. We also used multiple logistic regression modeling to estimate the degree of pharmacogenetic association of ‘specific’ AEs explained by the genotypes of the chosen SNPs. For this multivariate model, we considered adjustments for the following potential confounders: age, BMI, year since last menopause, year since the start of letrozole, and chemotherapy. The patterns of allelic association were statistically similar, as observed for the genotypes.

**Table 3:** Estimated odds ratios and 95% confidence intervals for letrozole-induced AEs according to various genetic models of inheritance.

SNP	Genetic model	Genotype & Allele	MS-AEs (N = 81)		VMSs (N = 57)		MS-AEs + VMSs (N = 33)		Any AEs (N = 105)	
			OR (95% CI)	P value	OR (95% CI)	P value	OR (95% CI)	P value	OR (95% CI)	P value
*rs7158782*	Co-dominant 1	*AA vs AG*	0.94 (0.49–1.77)	0.87	1.12 (0.56–2.20)	0.86	1.02 (0.43–2.26)	1.0	1.0 (0.56–1.84)	1.0
	Co-dominant 2	*AA vs GG*	0.83 (0.25–2.60)	0.78	0.19 (0.01–1.46)	0.14	0.33 (0.02–2.04)	0.43	0.64 (0.20–2.11)	0.57
	Dominant	*AA vs AG+GG*	0.92 (0.50–1.69)	0.87	0.94 (0.49–1.88)	1.0	0.89 (0.40–2.04)	0.84	0.94 (0.53–1.68)	0.88
	Recessive	*AA+AG vs GG*	0.85 (0.27–2.66)	1.0	0.18 (0.01–1.31)	0.15	0.33 (0.02–2.38)	0.44	0.64 (0.21–1.99)	0.58
	Over-dominant	*AA+GG vs AG*	0.96 (0.52–1.76)	1.0	1.26 (0.65–2.41)	0.64	1.12 (0.48–2.63)	0.83	1.06 (0.60–1.89)	0.88
	Additive		0.95 (0.55–1.59)	0.91	0.80 (0.44–1.46)	0.47	0.98 (0.57–1.70)	1.0	0.86 (0.46–1.64)	0.75
	Allele	*A vs G*	0.92 (0.56–1.48)	0.80	0.80 (0.47–1.40)	0.49	0.80 (0.42–1.50)	0.62	0.89 (0.56–1.40)	0.64
*rs7159713*	Co-dominant 1	*AA vs AG*	0.93 (0.49–1.77)	0.87	1.02 (0.50–2.0)	1.0	1.07 (0.45–2.37)	1.0	0.94 (0.51–1.72)	0.87
	Co-dominant 2	*AA vs GG*	0.82 (0.25–2.74)	0.78	0.19 (0.01–1.40)	0.14	0.34 (0.02–2.08)	0.43	0.62 (0.19–2.04)	0.57
	Dominant	*AA vs GG+AG*	0.91 (0.49–1.69)	0.87	0.85 (0.44–1.71)	0.73	0.93 (0.42–2.14)	1.0	0.87 (0.50–1.57)	0.66
	Recessive	*AG+AA vs GG*	0.85 (0.27–2.66)	1.0	0.18 (0.01–1.31)	0.15	0.33 (0.02–2.38)	0.44	0.64 (0.21–1.99)	0.58
	Over-dominant	*AA+GG vs AG*	0.96 (0.51–1.76)	1.0	1.14 (0.57–2.21)	0.72	1.18 (0.51–2.63)	0.68	0.98 (0.55–1.77)	1.0
	Additive		0.93 (0.53–1.74)	1.0	0.77 (0.41–1.44)	0.45	1.0 (0.58–1.75)	0.97	0.84 (0.44–1.57)	0.90
	Allele	*A vs G*	0.91 (0.55–1.48)	0.80	0.74 (0.42–1.27)	0.33	0.82 (0.43–1.54)	0.62	0.85 (0.53–1.35)	0.55
*rs2369049*	Co-dominant 1	*AA vs AG*	0.90 (0.45–1.74)	0.86	1.0 (0.48–2.01)	1.0	0.94 (0.41–2.13)	1.0	0.95 (0.51–1.76)	0.87
	Co-dominant 2	*AA vs GG*	1.12 (0.32–3.91)	1.0	0.25 (0.02–1.73)	0.25	0.44 (0.03–3.11)	0.67	0.86 (0.25–2.97)	1.0
	Dominant	*GG+AG vs AA*	0.93 (0.52–1.76)	0.87	0.88 (0.44–1.70)	0.73	0.86 (0.38–2.01)	0.83	0.93 (0.53–1.62)	0.88
	Recessive	*GG vs AG+AA*	1.16 (0.34–3.89)	1.0	0.25 (0.02–1.66)	0.25	0.45 (0.03–3.01)	0.67	0.87 (0.26–2.92)	1.0
	Over-dominant	*AA+GG vs AG*	0.89 (0.46–1.68)	0.74	1.08 (0.52–2.13)	0.86	1.0 (0.44–2.22)	1.0	0.95 (0.52–1.75)	1.0
	Additive		0.98 (0.57–1.70)	1.0	0.81 (0.48–1.33)	0.72	0.88 (0.47–1.66)	0.78	0.96 (0.53–1.82)	1.0
	Allele	*A vs G*	0.98 (0.60–1.60)	1.0	0.79 (0.45–1.42)	0.47	0.81 (0.40–1.62)	0.60	0.93 (0.58–1.49)	0.80
*rs11849538*	Co-dominant 1	*CC vs CG*	0.88 (0.46–1.64)	0.74	1.18 (0.60–2.28)	0.72	0.93 (0.39–2.03)	1.0	1.02 (0.56–1.83)	1.0
	Co-dominant 2	*CC vs GG*	0.95 (0.35–3.01)	1.0	0.20 (0.02–1.51)	0.15	0.32 (0.02–1.96)	0.43	0.76 (0.28–2.37)	0.78
	Dominant	*GG+CG vs CC*	0.89 (0.48–1.63)	0.76	1.0 (0.52–1.98)	1.0	0.82 (0.37–1.87)	0.68	0.97 (0.55–1.72)	1.0
	Recessive	*GG vs CG+CC*	1.0 (0.37–2.98)	1.0	0.19 (0.01–1.31)	0.15	0.33 (0.02–2.38)	0.44	0.75 (0.28–2.22)	0.79
	Over-dominant	*CC+GG vs CG*	0.88 (0.48–1.61)	0.75	1.32 (0.69–2.51)	0.49	1.02 (0.44–2.38)	1.0	1.05 (0.60–1.84)	0.88
	Additive		1.01 (0.59–1.78)	1.0	0.90 (0.51–1.62)	0.84	0.93 (0.53–1.73)	1.0	1.03 (0.37–2.76)	1.0
	Allele	*C vs G*	0.93 (0.58–1.48)	0.81	0.84 (0.48–1.43)	0.59	0.75 (0.40–1.48)	0.51	0.93 (0.60–1.45)	0.82

SNP, single nucleotide polymorphism; MS-AEs, musculoskeletal adverse events; VMSs, vasomotor symptoms; Both, patients with MS-AEs and VMSs, Any AEs, patients with either MS-AEs or VMSs; N, number of subjects; OR, odds ratio; CI, confidence interval; P, probability.

**Fig 1:**
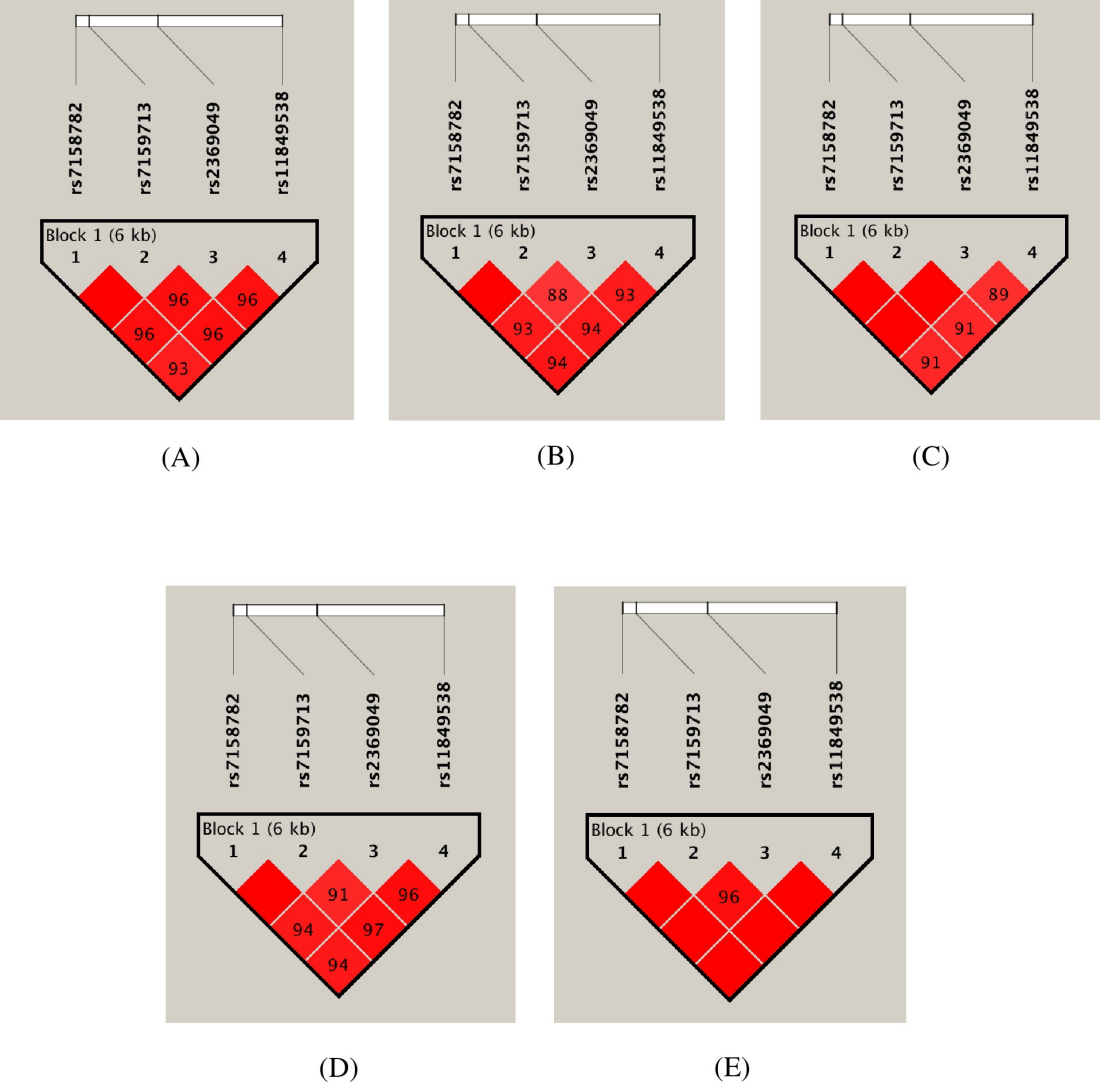
The figure shows the output of the Haploview LD pattern of *TCL1A* gene polymorphisms in patients with and without ‘specific’ AEs. Each square shows the pairwise LD relationship between two SNPs and the values inside the square denote D’. The color gradient from red to white reveals higher to lower LD for D’ (1–0). (A) Patients with MS-AEs, (B) Patients with VMSs, (C) Patients with MS-AEs and VMSs, (D) Patients with either MS-AEs or VMSs and (E) Patients without any AEs.

We also assessed the influence of *TCL1A* gene haplotypes with letrozole-induced ‘specific’ AEs. Fig 1 shows the linkage disequilibrium (LD) patterns of *TCL1A* gene polymorphisms. Overall, we observed high LD in all patient groups. Four marker haplotype analyses inferred four different haplotypes with a frequency of above 1%. There was no significant difference in the proportion of these haplotype frequencies between patients with and without ‘specific’ AEs (Table 4). Further, we have performed two-marker sliding window analyses, which also indicated similar outcomes (Table 5).

**Table 4:** Frequency distribution and association analyses of four marker haplotypes of *TCL1A* gene with letrozole-induced AEs.

Haplotypes	Haplotype composition				All	No AEs	MS-AEs	*χ2*	P	VMSs	*χ2*	P	Both	*χ2*	P	Any AEs	*χ2*	*P*
	*SNP1*	*SNP2*	*SNP3*	*SNP4*														
H1	A	*A*	*A*	*C*	72.2	72.0	72.2	0	0.96	74.5	0.22	0.63	75.7	0.34	0.55	72.4	0	0.94
H2	*G*	*G*	*G*	*G*	21.2	22.6	21.6	0.04	0.82	16.6	1.52	0.21	18.2	0.56	0.45	20.0	0.39	0.53
H3	*G*	*G*	*A*	*G*	3.3	3.8	2.5	0.47	0.49	3.5	0.01	0.90	3.0	0.07	0.78	2.9	0.25	0.61
H4	*A*	*A*	*A*	*G*	1.5	1.1	0	0.36	0.54	1.8	0.24	0.61	1.5	0.08	0.77	1.9	0.46	0.49

H, haplotypes; SNP1, rs7158782; SNP2, rs7159713; SNP3, rs2369049; SNP4, rs11849538; MS-AEs, musculoskeletal adverse events; VMSs, vasomotor symptoms; Both, patients with MS-AEs and VMSs; Any AEs, patients with either MS-AEs or VMSs; No AEs, patients without any AEs; χ2, chi-square; p, probability.

**Table 5:** *TCL1A* haplotypes in two-marker sliding window analyses and their association with ‘specific’ AEs.

*TCL1A* markers				All	MS-AEs	*χ2*	P	VMSs	*χ2*	P	Both	*χ2*	P	Any AEs	*χ2*	P
*SNP1*	*SNP2*	*SNP3*	*SNP4*													
*A*	*A*			74.2	74.7	0.11	0.73	77.2	0.62	0.43	77.3	0.43	0.50	75.2	0.23	0.63
*G*	*G*			24.7	24.7	0.12	0.72	21.1	1.07	0.29	22.7	0.33	0.56	23.3	0.48	0.48
*G*	*A*			1.0	-	-	-	1.8	1.05	0.30	-	-	-	1.4	0.78	0.37
	*A*	*A*		74.2	74.7	0.11	0.73	77.1	0.61	0.43	77.3	0.43	0.50	75.2	0.23	0.63
	*G*	*G*		21.4	22.2	0.06	0.93	17.5	1.09	0.29	19.7	0.23	0.62	20.4	0.25	0.61
	*G*	*A*		3.3	2.5	0.47	0.49	3.6	0.01	0.91	3.0	0.07	0.78	2.9	0.25	0.61
	*A*	*G*		1.0	-	-	-	1.8	1.02	0.31	-	-	-	1.5	0.75	0.38
		*A*	*C*	72.5	72.8	0.02	0.86	74.5	0.22	0.63	75.7	0.34	0.55	72.8	0.03	0.85
		*G*	*G*	22.2	22.2	0.04	0.84	18.3	0.93	0.33	18.1	0.69	0.40	21.4	0.16	0.68
		*A*	*G*	5.1	4.3	0.05	0.81	6.2	0.23	0.62	4.6	0	0.92	5.3	0.03	0.85

M, marker; MS-AEs, musculoskeletal adverse events; VMSs, vasomotor symptoms; Both, patients with MS-AEs and VMSs; Any AEs, patients with either MS-AEs or VMSs; χ2, chi-square; p, probability.

## Discussion

Breast cancer, a highly dreadful disease, is globally escalating [20]. It is often associated with poor prognosis and adverse outcomes. This is because of the striking variability in response to modern anti-cancer medications. Inter-individual differences underpinning pharmacological responses are driven for the most part by common genetic variants, the so-called polymorphisms in the DNA sequence of the genes encoding proteins, which controls drug absorption, distribution, metabolism, and excretion [21]. Over the decade, accelerated developments in molecular biology have brought a revolution in molecular medicine. One of the significant areas of biomarker research using pharmacogenomics as a screening tool is cancer.

As with most anti-cancer drugs, it is necessary to identify a potent pharmacogenetic marker that could determine the treatment outcomes of AIs. In addition, to a lesser extent, researchers investigated the role of *TCL1A* gene polymorphisms in the etiology of AIs mediated toxicity [15,22]. Genetic polymorphisms in the *TCL1A* loci gained attention after the indication of its association with MS toxicity risk in breast cancer patients undergoing anti-hormonal therapy with third-generation AIs [15]. Existing epidemiological investigations have implicated that race or ethnicity is an important factor inducing gene-drug relationships [23]. As of now, the normative frequency of these *TCL1A* gene polymorphisms was established only in post-genome project populations, namely HapMap (http://www.Hapmap.org) and 1000 genomes (http://www.1000Genomes.org). Nevertheless, more recently, we studied the distribution of four clinically important genetic polymorphisms in *TCL1A* gene loci and described significant inter-ethnic variation [19]. The variant allele frequency of the *TCL1A* gene differs by ethnicity. The SNPs namely, rs7158782, rs7159713, rs2369049, and rs11849538 were found to occur at a higher frequency of 75–84.2%, 75–84.1%, 73–84.1%, and 34–44.1%, respectively, in African populations, but were identified with very less frequencies in Asians (13.1–29%, 13.6–29%, 3.5–27%, and 17–30%, respectively), Caucasians (13.7–15.9%, 13.7–15.9%, 13.3–15.3%, and 8.8–14.0%, respectively), and Hispanics (21–25%, 21–25%, 13–24%, and 17–18%, respectively).

This may be the first study to evaluate the association of *TCL1A* gene variants in South Indians to the best of our knowledge and with the available evidence. We observed no significant SNP-level or haplotype-based associations of letrozole-induced ‘specific’ AEs for *TCL1A* genetic variants. Only two studies have been carried out to elucidate the possible role of *TCL1A* genetic polymorphisms on AI-associated outcomes [15,22]. A case-control GWAS identified four polymorphisms (rs7158782, rs7159713, rs2369049, and rs11849538) corresponding to the *TCL1A* gene at chromosome 14, which were attributed to increased MS-AEs [15]. In contrast, the other study explored the potential association between toxicity-related discontinuation of AI medication and genetic variants in the *TCL1A* gene. In that study, the authors unable to confirm the findings of GWAS in their prospective observation performed on breast cancer patients enrolled from Exemestane and Letrozole Pharmacogenetics (ELPH) clinical trial [22]. Notably, despite close LD between these polymorphisms, their impact on the occurrence of MS-AEs and VMSs could not be evaluated in our study, which supported the previous findings by Henry et al [22]. Furthermore, a significant interaction between *TCL1A* polymorphism (rs7158772) and drug effectiveness of AIs was not found in 308 women with metastatic breast cancer [24]. Similarly, in a study of anastrozole versus tamoxifen following five years of adjuvant use, the polymorphism rs11849538 was thought to be related to AIs efficacy; however, no association was found [12].

Moreover, the variant genotypes at SNP rs11849538 created an estrogen response element (ERE). Besides, this gene was found to be involved in the regulation of a series of genes encoding inflammatory response proteins (cytokines and its receptors) including the IL13RA1 (interleukin 13 receptor, a1), IL18R1 (interleukin 18 receptor 1), IL1R2 (interleukin 1 receptor, type 2), IL17RA (interleukin receptor A), and IL12RB2 (interleukin 12 receptor, b2). Subsequent in vitro experiments confirmed the estrogen-dependent transcription of the *TCL1A* gene; however, the mechanism by which the change in the *TCL1A* expression induces MS-AEs remained unclear [15]. However, recently, Ho and co-workers performed RNA-seq and Chip-seq experiments in the lymphoblastoid cell lines model system. They demonstrated the transcriptional factor role of the *TCL1A* gene and the molecular mechanism for SNP-estrogen-dependent regulation across the genome [25]. Additionally, it has also been reported that genetic variants in genes such as *RANKL* (rs7984870), *OPG* (rs2073618), and *ESR1* (rsrs2234693 and rs9340799) were associated with AI-related MS-AEs [26–28].

In this cohort, we could not evaluate the relationship between *TCL1A* variants and letrozole-induced ‘specific’ AEs. However, we cannot exclude the biological significance of *TCL1A* on patients’ risk of AEs. One of the possible explanations for this non-significant effect is the sample size. Our sample size (n = 198) for the association analyses was smaller than those in the existing studies [15,22]. The GWAS which discovered the significant role of *TCL1A* SNPs was primarily conducted in 878 subjects of white descent [15]. Importantly, there were no such significant associations observed in the following ELPH cohort involving 467 patients [22]. Other potential factors, including ethnicity, disease stage, and study design, could contribute to the mixed results among the studies. It should be noted that the current investigation has some limitations. First, we could not do a time-to-event analysis, which is one of the limitations of the study. Another limitation is that in the present study, the analytical plan did not envisage the association of genetic variation with some grades of AEs. Although the study has enough power to reflect the probability of a statistical meaning, we cannot exclude the possibility of false-negative outcomes due to the small sample size; thus, the results for these analyses should be interpreted with caution.

## Conclusions

In summary, we found no statistically significant association between the four studied *TCL1A* SNPs and letrozole-induced ‘specific’ AEs. Thus, it appears unlikely that polymorphisms in *TCL1A* loci may be a robust predictor for AEs in HR+ breast cancer patients treated with letrozole. However, in order to further refine the role of these *TCL1A* genetic variants on the etiology of AI-induced AEs, future studies should include a greater number of patients sample for analysis. Furthermore, future investigations could systematically examine the interactive effects (epistatic) between candidate genes and AI-induced AE.
